# Community Engagement to Improve Equity in Kidney Transplantation from the Ground Up: the Southeastern Kidney Transplant Coalition

**DOI:** 10.1007/s40472-021-00346-x

**Published:** 2021-10-31

**Authors:** Rachel E. Patzer, Samantha Retzloff, Jade Buford, Jennifer Gander, Teri Browne, Heather Jones, Matt Ellis, Kelley Canavan, Alexander Berlin, Laura Mulloy, Eric Gibney, Leighann Sauls, Dori Muench, Amber Reeves-Daniel, Carlos Zayas, Derek DuBay, Rich Mutell, Stephen O. Pastan

**Affiliations:** 1 Department of Surgery, Emory University School of Medicine, Atlanta, Georgia; 2 Department of Epidemiology, Rollins School of Public Health, Emory University, Atlanta, Georgia; 3 Center for Research and Evaluation, Kaiser Permanente Georgia, Atlanta, Georgia; 4 College of Social Work, University of South Carolina, Columbia, South Carolina; 5 Vidant Medical Center, Greenville, North Carolina; 6 Department of Medicine and Surgery, Duke University, Durham, North Carolina; 7 National Kidney Foundation, New York NY; 8 Southeastern Kidney Transplant Coalition patient partner; 9 Division of Nephrology, Department of Medicine, Augusta University, Augusta, Georgia; 10 Piedmont Healthcare, Piedmont Transplant Institute, Atlanta, Georgia; 11 Alliant Quality Improvement Services Group, Alliant Health Solutions, Raleigh, North Carolina; 12 Abdominal Organ Transplant Program, Wake Forest Baptist Health, Winston Salem, North Carolina; 13 Department of Internal Medicine, Wake Forest School of Medicine, Winston Salem, North Carolina; 14 Division of Nephrology, University of South Carolina Keck School of Medicine, Columbia, South Carolina; 15 Department of Surgery, College of Medicine, Medical University of South Carolina, Charleston, South Carolina; 16 Apex Health Innovations; 17 Renal Division, Department of Medicine, Emory University School of Medicine, Atlanta, Georgia

**Keywords:** transplant, access, population health

## Abstract

**Purpose of review:**

The purpose of this review is to describe the Southeastern Kidney Transplant Coalition’s mission, vision, goals, and Early Transplant Access registry as an example of a community/academic collaboration dedicated to improving access to transplantation and reducing inequities in transplant access.

**Recent findings:**

The barriers and facilitators to referral and evaluation for kidney transplantation are not necessarily the same as for waitlisting and transplantation. Recent findings suggest that inequities in transplant access are multilevel and multifactorial and require continued community engagement to improve access to kidney transplantation across patients, health systems, and populations.

**Summary:**

Community-engaged approaches are critical to ensuring that inequities in transplant access – which may vary across regions -- are not only described but are addressed in practice in a sustainable manner.

## Introduction

Kidney transplantation is the optimal treatment for end-stage kidney disease patients and the process to receive a transplant involves multiple required steps(1). Substantial variation in access to transplantation has been observed across geographic regions and dialysis facilities (2–4). In 2010, when the Southeastern U.S. was identified as having the lowest rates of transplant in the nation (2), stakeholders in the kidney disease community collaborated to form the Southeastern Kidney Transplant (SEKTx) Coalition. For over ten years, the SEKTx Coalition has focused grassroots quality improvement and research efforts on improving access to kidney transplant and reducing disparities, especially at the early steps in the transplant process.

While the majority of quality improvement and research efforts in kidney transplantation have focused on access to the waiting list, transplant, and post-transplant outcomes, the SEKTx Coalition has focused on transplant steps prior to waitlisting, including referral and evaluation for transplant, and on connecting the disparate health systems involved in transplant access. For example, dialysis units play a key role in early access to transplant because the majority of kidney failure patients start treatment on dialysis (1, 5), and facilities are mandated by Centers for Medicare and Medicaid (CMS) and End-Stage Renal Disease (ESRD) Networks to educate patients on transplant and help patients navigate the many steps in the transplant process necessary to get on the transplant waiting list. However, disparities in access to transplant have been observed at the dialysis-facility level, and the many patients who are not pre-emptively waitlisted for transplant rely on healthcare professionals in dialysis settings to refer them for transplant evaluation. Thus, the SEKTx Coalition coalesced multidisciplinary stakeholders and community members to address the many inequities in access to transplant. This papers reviews the mission, vision, goals, and community activities of the SEKTx Coalition in the context of recent Coalition findings about the growing need for improving equity in access to early steps of kidney transplantation. We then identify opportunities for improvement and change to improve transplantation parity in the United States.

## History of the SEKTx Coalition: Mission, Vision, and Goals

**Figure 1.**
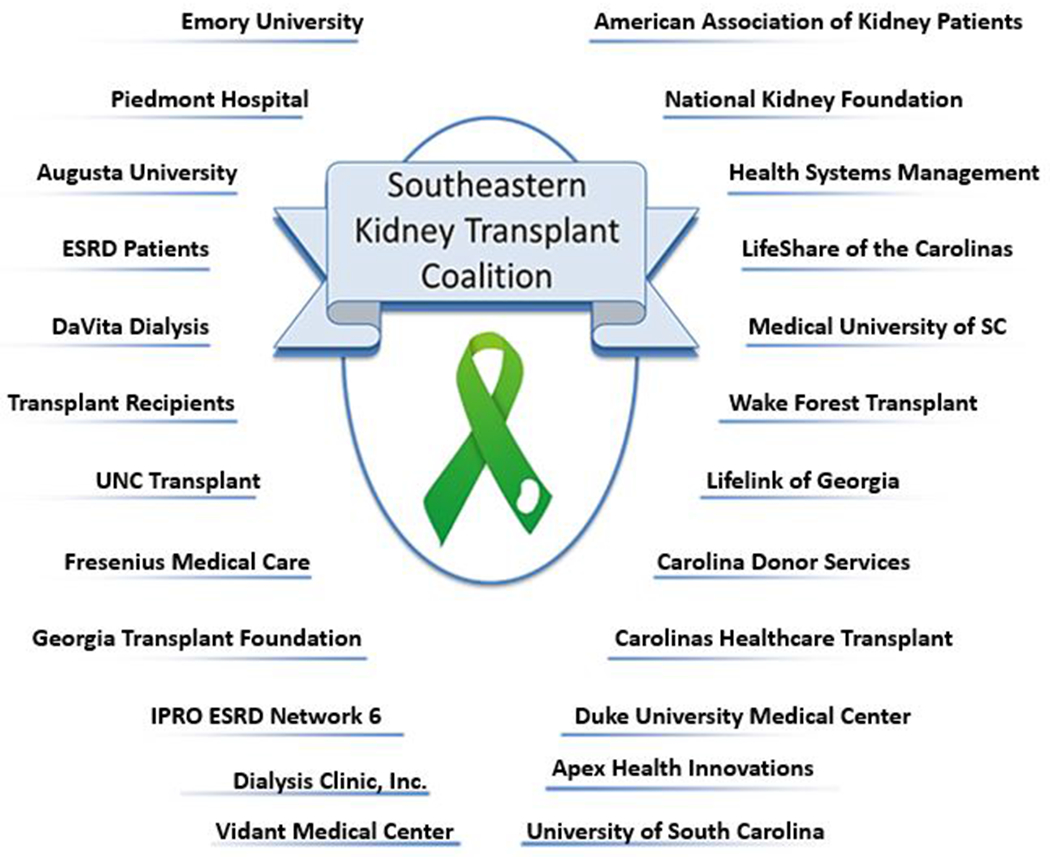
The Southeastern Kidney Transplant Coalition Stakeholders and Partnering Organizations in Georgia, North Carolina, and South Carolina.

The SEKTx Coalition was formed in 2010 and was initially motivated by an ESRD Network 6 (Georgia, North Carolina, South Carolina) quality improvement project focused on increasing regional transplant rates and reducing barriers to transplantation. There are 18 geographically-defined ESRD Networks in the United States, each mandated by CMS to coordinate with ESRD patients, dialysis and transplant providers, and community partners to improve patient care. Using a community-based approach to work towards the CMS and ESRD Network 6 goal of identifying and reducing barriers to transplant, the SEKTx Coalition was formed with a mission of increasing kidney transplant rates by identifying and reducing barriers in the kidney transplant process and a vision of improving equity in access to kidney transplantation for kidney disease patients in the Southeast region. The Coalition consists of voluntary stakeholders, including patients, family members, care partners, transplant centers, health care professionals, organ procurement organizations, academic researchers, patient advocacy groups, representatives of large and small dialysis companies, and ESRD Network 6 staff (Figure 1). Together, the SEKTx Coalition agreed upon goals to: 1) increase dedication and awareness among kidney disease patients, providers, and the public; 2) build an alliance of transplant centers; 3) increase the availability of organs; and 4) identify, test, and distribute best practices in the region.

While the geographic variability in kidney transplant rates have been well-known for decades,(4) the initial formation of the SEKTx Coalition was primarily motivated by data that showed ESRD Network 6 of GA, NC, and SC had the lowest rates of kidney transplantation of all 18 ESRD Networks (2, 6). At the initial SEKTx Coalition kick-off meeting in 2011, the group members reviewed literature and offered wide-ranging hypotheses to hypothesize the patient-, dialysis facility-, provider-, neighborhood, ESRD Network- and health system-level factors that may be associated with comparatively low access to kidney transplant for patients in the Southeast.(4, 7–10) Following formation of the group, SEKTx Coalition partners conducted a needs assessment of the ESRD patient population in GA, NC, and SC to identify gaps in understanding and to inform and conduct quality improvement projects and interventions aimed at increasing and improving equity in access to transplant.

As part of the quantitative needs assessment, SEKTx Coalition members examined the dialysis facility characteristics and ESRD Network factors that contribute to low transplant rates.(2, 11) Results showed that barriers to accessing kidney transplant for patients in the Southeast were multi-level (e.g., occurring at patient, provider, neighborhood, and health system levels) and multi-factorial (2, 6, 11). For example, dialysis facilities having a higher percentage of black patients, a higher percentage of patients without healthcare coverage, and patients with more comorbidities, were associated with decreased facility-level transplant rates (2). Surveys of dialysis facility staff that SEKTx Coalition members (ESRD Network staff and researchers) led found that while the vast majority of staff felt comfortable discussing transplant with their patients, only 19% thought that >50% of their patients were interested in transplant (12), while >70% of ESRD patients self-reported interest in transplant (13). Further, dialysis patients who participated in three different SEKTx Coalition focus groups, led by ESRD Network staff, researchers, and staff from the National Kidney Foundation, in GA, NC, and SC discussed barriers to kidney transplant revealing two novel themes: the need for increased and meaningful provision of transplant education by dialysis facility staff, and the importance of encouragement and navigation through the transplant process by dialysis facility staff (13).

Results of the Coalition’s needs assessment and primary qualitative and quantitative data collection demonstrated the importance of factors at multiple levels that contribute to disparities in access to transplant in the Southeast, including dialysis facility characteristics (2) and transplant practices (12), dialysis provider perceptions and potential biases of their patients and the transplant process (12, 13), and socioeconomic factors such as neighborhood characteristics and upstream social determinants of health (11). Thus, a multi-component quality improvement intervention targeting barriers to transplant at multiple levels was recommended by the SEKTx Coalition, with a focus on dialysis facilities and patient populations with historically lower access to transplant (14). The SEKTx Coalition members also recognized important gaps in knowledge about the factors contributing to low kidney transplantation rates and inequities in access to transplantation, especially among black ESRD patients in the Southeastern region. This work helped to inform the future quality improvement and research projects that the SEKTx Coalition prioritized to meet its mission, vision, and goals.

## Collaborative SEKTx Coalition projects aimed at increasing access and reducing disparities in access to transplant

To meet the mission, vision, and goals of the SEKTx Coalition, members have typically met annually in-person and quarterly via conference call and have collaborated on multiple quality improvement projects, pragmatic research trials of multi-component interventions both regionally and nationally, scientific manuscripts, conference presentations, policy initiatives to improve equity in access to transplantation, and community dissemination of information about transplant access and inequities. The SEKTx Coalition has developed and tested several multicomponent interventions that have been implemented to improve access to transplantation. The first intervention that the SEKTx members led was the Reducing Disparities in Access to Kidney Transplantation (RaDIANT) Community Study, a quality improvement and research study, piloted in GA in 2013, the state with the lowest rates of kidney transplantation among all ESRD patients at that time (15, 16). The multilevel RaDIANT intervention was developed and delivered among 134 facilities in Georgia. The dialysis facilities, which served more than 9,000 patients, were randomized to receive either an intervention consisting of transplant education and engagement tasks targeting dialysis facility leadership, dialysis staff, and dialysis patients, or the standard-of-care, “usual” transplant education provided at dialysis facilities (15).

The intervention activities were co-developed with stakeholder members of the SEKTx Coalition and included existing resources and infrastructure from members, intended to ensure sustainability of intervention activities over time. For example, several existing intervention materials that had either known efficacy, high face and content validity, and were freely available were included as components of the RaDIANT multicomponent interventions. In order to facilitate increased ESRD patient knowledge about the option of kidney transplantation and to encourage shared decision-making with their providers, a mobile clinical decision aid and website called iChoose Kidney was included to aid dialysis providers in comparing individualized patient mortality risk estimates for dialysis vs. transplant as treatment options (17, 18). Further, with input from Coalition members, Living ACTS (About Choices in Transplantation and Sharing) was developed by one of the research teams of a SEKTx Coalition member to act as a culturally-sensitive educational tool designed to address barriers to living donor kidney transplantation among black ESRD patients (19, 20). Other existing resources by community partners were also leveraged, such as a dialysis peer mentoring program by the Georgia Transplant Foundation, one of the SEKTx Coalition partners (21), and SEKTx Coalition volunteers offered time to contribute expertise to monthly webinars to educate dialysis leadership and staff about various resources available to assist patients and facility staff with facilitating transplant access.(15, 16)

ESRD Network 6 staff were critical to the core components of the quality improvement interventions and regular communications with dialysis facility contacts. The large community-based participatory research RaDIANT project was only possible due to the aligned interests of these with the CMS Statement of Work for ESRD Networks that explicitly called for a project to improve transplant access and reduce transplant disparities (22). Additional support for study design, data collection, data analysis and scientific support was funded by a community-based participatory research grant from the National Institutes of Minority Health and Health Disparities. After 12 months of intervention activities, dialysis facilities that received the interventions referred a higher proportion of their ESRD patients compared to control facilities, and these facilities were more effective at improving the proportion of black (vs. white) patients referred, thereby improving equity in access to transplant referral in Georgia (16). The SEKTx Coalition members used findings from both the effectiveness and process evaluations from the RaDIANT Community study to assess what was successful and what intervention components were sustainable and possible to implement beyond one state (23). In 2018, the RaDIANT community intervention was adapted and expanded regionally to 440 dialysis facilities in GA, NC, and SC and included several of the same intervention components as well as new interventions focused on improving access and reducing disparities in the transplant center medical evaluation(24). For example, SEKTx Coalition partners Apex Health Innovations Inc. worked collaboratively with members to develop the Transplant Referral Exchange (T-REX) application, which allows electronic communication and transmission of patient transplant referrals between participating dialysis facilities and transplant centers (25).

Within the mission of improving equity in access to transplantation, the SEKTx Coalition has also collaborated on large scale quality improvement and research interventions on a national level. In response to the kidney allocation system (KAS) changes implemented by the United Network for Organ Sharing in 2014 to reduce disparities (26), the SEKTx Coalition helped to lead the Allocation System for Changes in Equity in Kidney Transplantation (ASCENT) study across all 18 participating ESRD Networks (27). The ASCENT study was co-designed by SEKTx Coalition members as well as a national Dissemination Advisory Board to extend the reach of the KAS policy by educating dialysis facility staff and their patients about how KAS may impact dialysis patients. The specific focus targeted minorities and patients who were good transplant candidates but were not yet referred for transplant, and would benefit from changes in the allocation system by going to the top of the waiting list if successfully referred, evaluated, and waitlisted. The primary goals of ASCENT were to improve access to the transplant waiting list, reduce disparities in waitlisting, and increase provider and patient knowledge of policy changes to improve access to transplantation (27, 28). The study was designed as an effectiveness-implementation pragmatic study design so that intervention materials -- a webinar for medical directors and facility staff, a 10-minute educational video targeting dialysis staff, a 10-minute educational video targeting patients, and a facility-specific audit and feedback report of transplant performance -- could be rapidly disseminated across a large population in need while simultaneously assessing the effectiveness of the multicomponent intervention.(27) While longer term outcomes are still being examined for this study, the interim outcome of dialysis provider knowledge about KAS was significantly improved three months after baseline among the intervention vs. control group (28). Among 655 U.S. dialysis facilities with low waitlisting nationwide included in ASCENT, baseline data showed that only 57.9% of dialysis facility staff were aware of the KAS change (29) and only 19% of staff were aware there was a racial disparity in waitlisting (29). After 3 months, knowledge scores among dialysis providers in the intervention were higher (mean difference in knowledge: 0.25 (95% confidence interval CI, 0.11-0.48; *P* = 0.039) compared to nonintervention providers, suggesting that dialysis facility provider education may extend the impact of a national policy change in kidney allocation and disparity reduction (28). The SEKTx Coalition’s co-development and co-implementation of multicomponent interventions demonstrates the feasibility and importance of community-engaged and collaborative approaches to increasing and improving equity in kidney transplantation.

## Access to Kidney Transplantation – The Early Kidney Transplant Access Registry

In addition to identifying dialysis facility-, provider-, and patient-level factors that may impact access to and disparities in kidney transplant, and the development and implementation of several multicomponent interventions, SEKTx Coalition members have also sought to address the gap in knowledge about disparities and variation in access to early steps of the transplant process. Variation in transplantation has been previously reported across dialysis facilities and geographic regions based on standardized transplantation ratios (2), and one regional study conducted by ESRD Network 8 more than 15 years ago reported variation in early transplant steps (7). However, most of the research focused on increasing kidney transplantation rates and eliminating disparities has focused on access to kidney transplant and the transplant waitlist. The SEKTx Coalition has long acknowledged that the lack of national surveillance data on transplant steps prior to patient placement on a waiting list, including referral and evaluation for transplant, limits the ability to effectively address inequities in transplant access(30). The significant disparities in transplant access that persist across geographic regions (2–4, 31), socioeconomic status, race/ethnicity, and sex (32, 33) may be difficult to address without understanding these early transplant steps.

**Figure 2.**
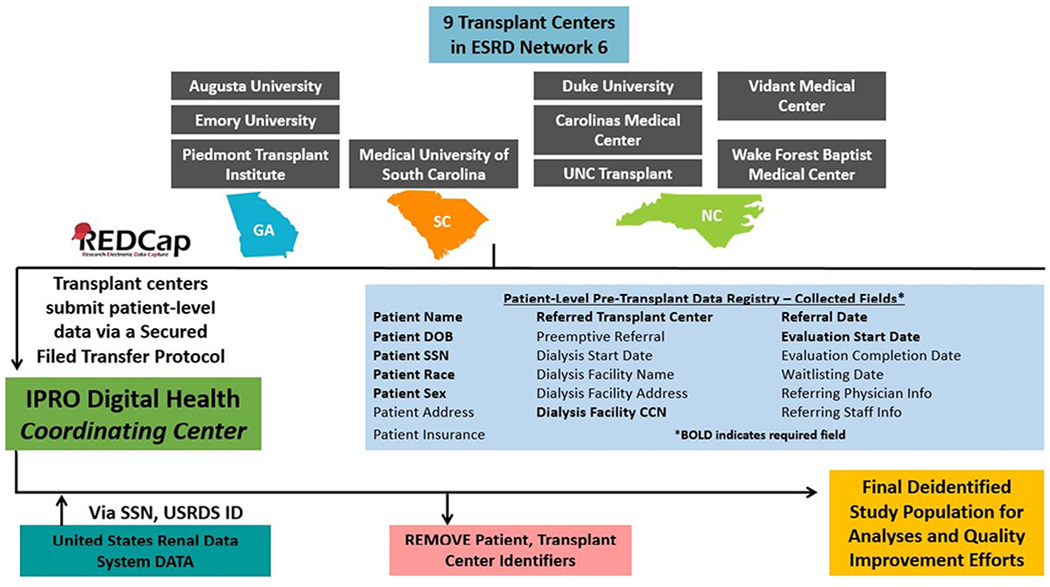
Early Transplant Access Data Registry Data Collection from SEKTx Coalition and ESRD Network 6 Transplant Centers (N=9) in Georgia, North Carolina, and South Carolina.

With no national data collection on pre-waitlisting steps of the transplantation process, limited information about transplant referral and evaluation (34, 35), and research showing that there are multi-level factors that may impact transplant access upstream in the process, the SEKTx Coalition established an Early Transplant Access data registry within the tristate region (Figure 2). Data collection on kidney transplant referral and evaluation began in 2012 in Georgia (34) and then expanded to include nine transplant centers in GA, NC, and SC (35). With a focus on learning more about access to early steps of the kidney transplant process in regions and patient populations outside of the Southeast, and to be able to conduct geographic comparisons, the SEKTx Coalition is currently expanding the collection of referral and evaluation data, targeting 48 transplant centers across 13 states: Connecticut, Georgia, Indiana, Kentucky, Maine, Massachusetts, New Hampshire, New York, North Carolina, Ohio, Rhode Island, South Carolina, and Vermont; thus far 30 transplant centers have voluntarily submitted data to ESRD Networks 1, 2, 6, and 9 which serve as the data coordinating center for these data.

**Figure 3.**
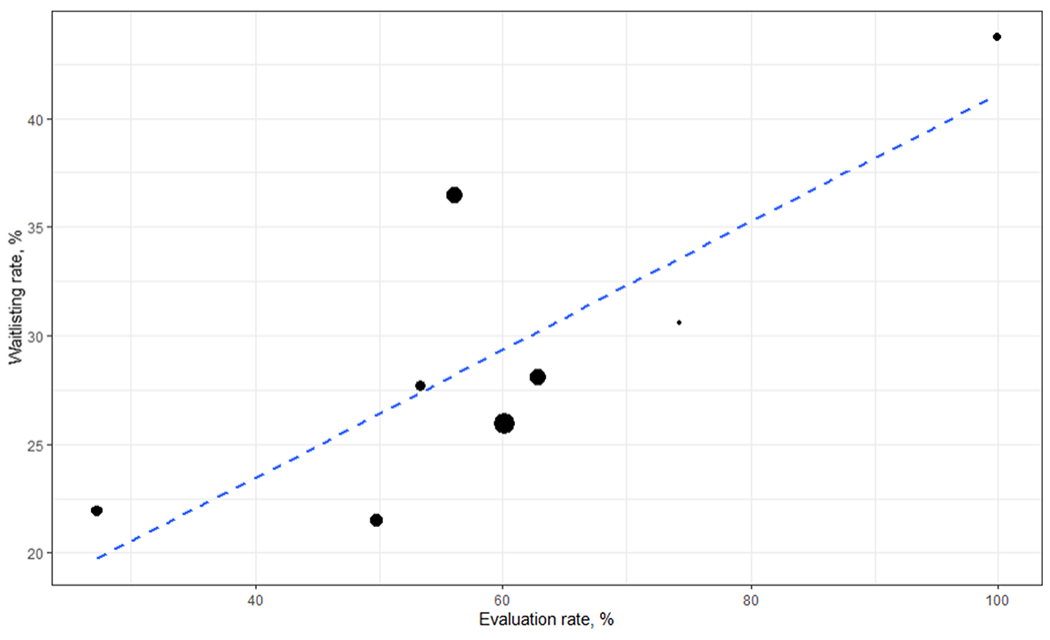
Variation in early transplant access among transplant programs in the Southeast, 2017-2020. Transplant evaluation rate (among referred patients) and the waitlisting rate (among evaluated patients) for 8 transplant programs in the Southeastern United States, demonstrating substantial variation in each outcome across transplant programs. Among transplant programs, substantial variation also exists in the proportion of patients evaluated within a year among those referred to a program (range: 27% to 99%) and among the proportion of patients waitlisted within a year of evaluation (range: 6.0% to 43.7%)

The feasibility and importance of national collection of these data has been further demonstrated through use of these data for research, quality improvement projects (including using referral and evaluation as outcome measures for ESRD Network quality improvement projects), pragmatic research trials, and the development of new quality metrics aimed at furthering knowledge about access and disparities in transplant at early steps in the process (36, 37). These data have demonstrated for the first time substantial variation in transplant referral and evaluation across dialysis facilities in the Southeast. Among ~35,000 ESRD patients starting dialysis in 2012-2016 in GA, NC, and SC, the median within-dialysis facility proportion of ESRD patients referred within 1 year was 33.7% and fewer than half of referred patients started the evaluation within 6 months of referral (35). Among transplant programs represented in the SEKTx Coalition, substantial variation also exists in the proportion of patients evaluated within a year among those referred to a program (range: 27% to 99%) and among the proportion of patients waitlisted within a year of evaluation (range: 6.0% to 43.7%) (Figure 3). Evidence from these studies in the Southeast also suggests that the socioeconomic factors associated with referral for transplant differ from factors associated with the start of the transplant evaluation (24, 35). For example, dialysis patients being treated at for-profit dialysis facilities in the Southeast were less likely to be referred for transplant, although there was no meaningful difference in patients starting transplant evaluation between those receiving care at for-profit vs. non-profit facilities (38).

Additionally, our SEKTx Coalition members have used data from the Early Transplant Access Registry for quality improvement and research to help support interventions to address inequities in transplant access. For example, the Coalition has helped ESRD Network 6 select dialysis facilities for a quality improvement intervention based on low rates of referral (16), and used these data as process measure outcomes for pragmatic trial interventions (e.g., RaDIANT and ASCENT) (16, 28). They have also used these data to provide feedback reports for transplant programs to monitor transplant referral and evaluations from dialysis facilities in their region.

The SEKTx Coalition’s Early Transplant Access Registry collection of transplant referral and evaluation data could have implications for transplantation and dialysis facility quality metrics. To date, kidney transplant program performance has typically been measured by post-transplant outcomes but has left out pre-transplantation measures due to the lack of national surveillance data. The lack of national-level data collection on waitlisting start and decisions limits the ability to investigate the disparities, quality, and identify best practices for improvement (39). Many patients who are eligible for kidney transplantation never reach the crucial steps of evaluation and are not captured within national data on transplantation (40). While there has been more of a recent emphasis on the average time spent on the waitlist with the inclusion of metrics for transplant rate and waitlist mortality for transplant centers in Scientific Registry of Transplant Recipients (SRTR) program-specific reports (36), these metrics could be improved with referral and evaluation start data.

To provide a more comprehensive understanding of transplant program performance and provide additional information to inform patient decisions, the SEKTx Coalition used data from the Early Transplant Access Registry to propose a new quality measure for transplant programs, the waitlisting rate (36). Our research found that centers that have high transplant rates do not necessarily have high waitlisting rates and vice versa. Additionally, the results demonstrate that the existing transplant rate quality metric used to determine the proportion of patients who are waitlisted misses the inclusion of important steps prior to waitlisting, including referral and evaluation. Given that there are many transplant centers and patient factors that are uncontrollable by dialysis facilities, it may be more relevant to measure quality based on earlier steps in the transplantation process, such as referral. Using our Early Transplant Access data registry, we also developed a new quality measure for dialysis facilities called the Standardized Transplant Referral Ratio (37), which could serve as pilot data for a measure that CMS could implement in practice (41).

## Collaborative and community-engaged approaches in research and quality improvement are key to improving access to transplant

The persistence of disparities in access to transplantation despite multi-level, community-based interventions focused on the waitlisting list, transplant, and post-transplant outcomes emphasizes the need for increased national focus on early transplant steps and identification of the multi-level barriers contributing to disparities in access to transplant. The persistence of disparities stresses the need for nationally collected data on referral and evaluation for transplant, which the SEKTx Coalition has demonstrated, is feasible using a community-engaged approach. In order to create pragmatic interventions to reduce current and future disparities in transplantation and improve access, there is a need to not only describe variation and barriers in access to early transplant steps in particular regions, but also to collaborate with ESRD community members, quality improvement organizations, patients, providers, and patient advocacy groups to act now to address these inequities. Many of the reasons for disparities in access to transplant will vary by region, necessitating active community engagement in directing resources and solutions to the problem. Recent policy directives have focused on increased and equitable access to transplants, indicating that it is time to advocate for increased attention on upstream steps within the transplant process to reduce disparities and ensure equity. In 2018, CMS directed ESRD Networks to focus quality improvement efforts not only on increasing the overall proportion of ESRD patients receiving transplants, but also to reduce racial and socioeconomic disparities in patient’s access to transplant.

Moreover, in 2019, the Advancing American Kidney Health initiative was announced, proposing an increase in the percent of eligible patients on a transplant wait-list to 30% by 2023, ensure that dialysis treatment options are more largely available for patients, and increase the number of available organs for transplantation (42). The SEKTx Coalition adopted a community-engaged, collaborative approach to increasing access to kidney transplant for kidney disease patients in the Southeast and sets an example for how we as an interdisciplinary kidney disease community can develop and implement interventions, address gaps in knowledge about access to transplant through novel data collection and mixed-methods research, and improve quality of patient care. The SEKTx Coalition has demonstrated the feasibility of national data collection on early transplant steps through the creation of the Early Transplant Data Registry and expansion to other regions in the United States. Furthermore, with increased national attention and policy directives focused on equity in access to kidney transplantation and steps prior to waitlisting, the SEKTx Coalition continues to advocate for nationally collected surveillance data on these important measures in the transplantation process. In addition to a focus on early transplant steps to ensure equitable access to the waiting list, the SEKTx Coalition also has specific interest in efforts to improve organ supply, including through decreasing organ discards as well as improving access to living donor transplantation.

## Conclusion

The SEKTx Coalition has fostered collaboration between interdisciplinary volunteers within the kidney disease community in the Southeastern U.S. to conduct a variety of community-engaged initiatives to improve access to kidney transplantation and reduce disparities. In research and quality improvement efforts, community-engaged approaches are critical to ensuring that inequities in transplant access are not only described but are addressed in practice and real-world settings. Community-based collaboration, leadership and funding support from at academic institutions and the ESRD Network have been critical to the formation and sustainability of the SEKTx Coalition. Community-based coalitions, such as the SEKTx Coalition, have the potential to improve access to kidney transplantation and reduce inequities at every step in the process.
